# Study on the influence of fixed dental prostheses on radiotherapy dose distribution based on dual-layer detector spectral CT imaging

**DOI:** 10.3389/fonc.2026.1910767

**Published:** 2026-09-10

**Authors:** Haoxin Pan, Lin Lu, Yudian Mao, Jianzhao Ni, Yujie Cao

**Affiliations:** 1 Department of Stomatology, The First Affiliated Hospital, Fujian Medical University, Fuzhou, China; 2 Department of Stomatology, National Regional Medical Center, Binhai Campus of the First Affiliated Hospital, Fujian Medical University, Fuzhou, China; 3 Key Laboratory of Radiation Biology of Fujian Higher Education Institutions, The First Affiliated Hospital, Fujian Medical University, Fuzhou, China; 4 Department of Radiotherapy, Cancer Center, The First Affiliated Hospital of Fujian Medical University, Fuzhou, China; 5 Stomatological Key laboratory of Fujian College and University & Institute of Stomatology & Research Center of Dental and Craniofacial Implants, School and Hospital of Stomatology, Fujian Medical University, Fuzhou, China

**Keywords:** artifact, dose distribution, electron density, fixed dental prostheses, radiotherapy

## Abstract

**Objective:**

Dental prostheses are often removed before radiotherapy due to metal-induced artifacts, impairing patients’ quality of life. This study evaluated five non-metallic materials for fixed prostheses in head and neck radiotherapy.

**Methods:**

Full crowns made of zirconia, lithium disilicate glass-ceramic, resin-matrix ceramic, PEEK, and PMMA were placed on an *in vitro* model with natural teeth as a control. Spectral CT scanning with and without artifact reduction was performed. Dose distributions for 6 MV and 10 MV photon beams were calculated using convolution superposition.

**Results:**

Artifact reduction significantly altered dose distributions for all materials (P < 0.001). Without artifact reduction, only zirconia differed from natural teeth (P < 0.001). After artifact reduction, no material showed significant difference from the control. Artifact-induced dose error was largest for zirconia, followed by resin-matrix ceramic and glass-ceramic, while errors for PEEK and PMMA were negligible.

**Conclusion:**

Zirconia is unsuitable due to severe artifacts. PEEK and PMMA are recommended for provisional crowns. Lithium disilicate glass-ceramic and resin-matrix ceramic are suitable for definitive restorations in radiotherapy patients.

## 1 Introduction

Head and neck cancer (HNC) accounts for over 800,000 new cases worldwide annually. Radiotherapy is a key treatment modality that requires precise dose distribution calculation using a treatment planning system (TPS) to eliminate tumors while protecting normal tissues. However, most middle-aged and elderly patients have high-density metal restorations (e.g., porcelain-fused-to-metal crowns) in their oral cavity. These cause severe artifacts on CT images, leading to dose enhancement at the entrance surface and dose attenuation at the exit surface (1). This not only increases damage to normal tissues but also results in insufficient dose to the target volume, compromising radiotherapy planning and delivery. Consequently, radiation oncologists often request the removal of fixed prostheses, which severely reduces patients’ quality of life, especially in those who have already undergone jaw resection due to cancer. Precision radiotherapy demands accurate positioning, planning, and treatment. Among TPS algorithms, clinically the collapsed cone convolution (CCC) algorithm is commonly used due to its higher efficiency and minimal difference from Monte Carlo (MC) (2).

Recent RayStation-based studies further support the clinical relevance of evaluating treatment plans using commercial treatment planning system workflows. El Mouden et al. assessed head-and-neck VMAT planning in RayStation by comparing multicriteria optimization with standard optimization, combining dosimetric plan-quality evaluation with delivery accuracy analysis (3). In addition, another recent RayStation study evaluated 6 MV FF and FFF beam modes for pediatric craniospinal irradiation, focusing on both planning quality and treatment deliverability (4). These studies highlight the importance of assessing dose calculation and plan quality under clinically relevant treatment-planning conditions. Therefore, the present study used a RayStation-based TPS workflow with the CCC algorithm to evaluate the dosimetric impact of different fixed dental prosthetic materials.

Existing studies have mostly focused on metallic materials, with insufficient attention to non-metallic materials, and most have not excluded the interference of artifacts on dose calculation. Spectral techniques combined with metal artifact reduction algorithms can effectively remove artifacts, such as with dual-layer detector spectral CT (IQon Spectral CT) (5). Modern linear accelerators commonly use 6 MV and 10 MV X-rays; energies above 10 MV tend to produce neutron contamination. Recently, various non-metallic dental materials with low density, low artifact, and good biocompatibility have been developed (e.g., zirconia, lithium disilicate, resin-matrix ceramics, PEEK), making them potential candidates for ideal fixed prostheses in radiotherapy patients.

Objective of this study: To simulate a real oral radiotherapy environment for patients with fixed prostheses, acquire artifact-containing and artifact-reduced images using dual-layer detector spectral CT, calculate dose distributions using the CCC algorithm under 6 MV and 10 MV photon beams, compare the impact of different non-metallic fixed prosthetic materials on dose distribution in radiotherapy plans, and evaluate dose differences before and after artifact reduction, in order to identify suitable fixed prosthetic materials for head and neck cancer patients undergoing radiotherapy.

## 2 Materials and methods

### 2.1 Collection of extracted teeth

Three morphologically intact extracted teeth—one mandibular second premolar, one mandibular first molar, and one mandibular second molar—were collected. The donors were adults aged 50 ± 10 years, and teeth were extracted due to periodontal disease.

### 2.2 Fabrication of study model

**Figure 1:**
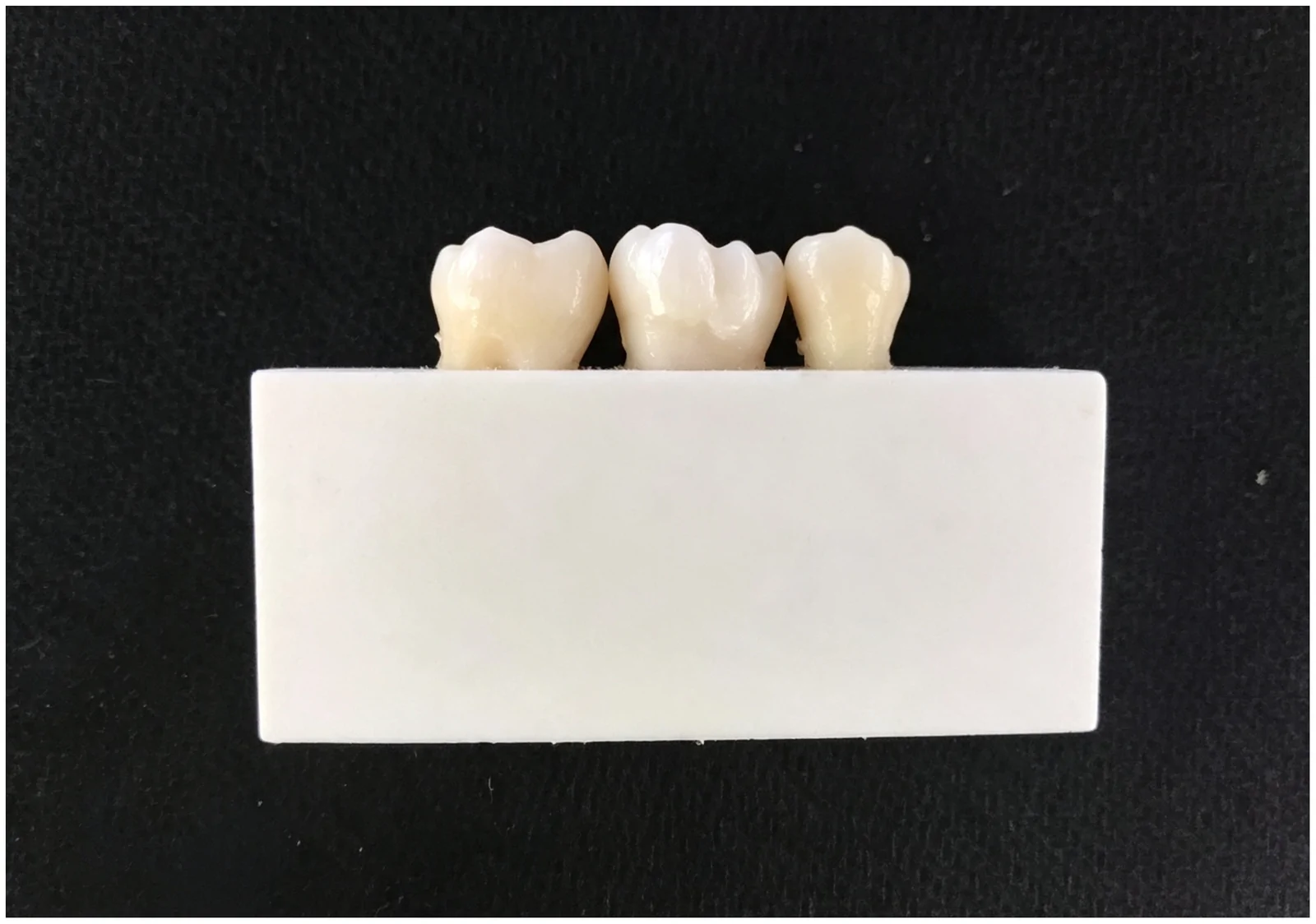
Partial mandible model.

**Figure 2:**
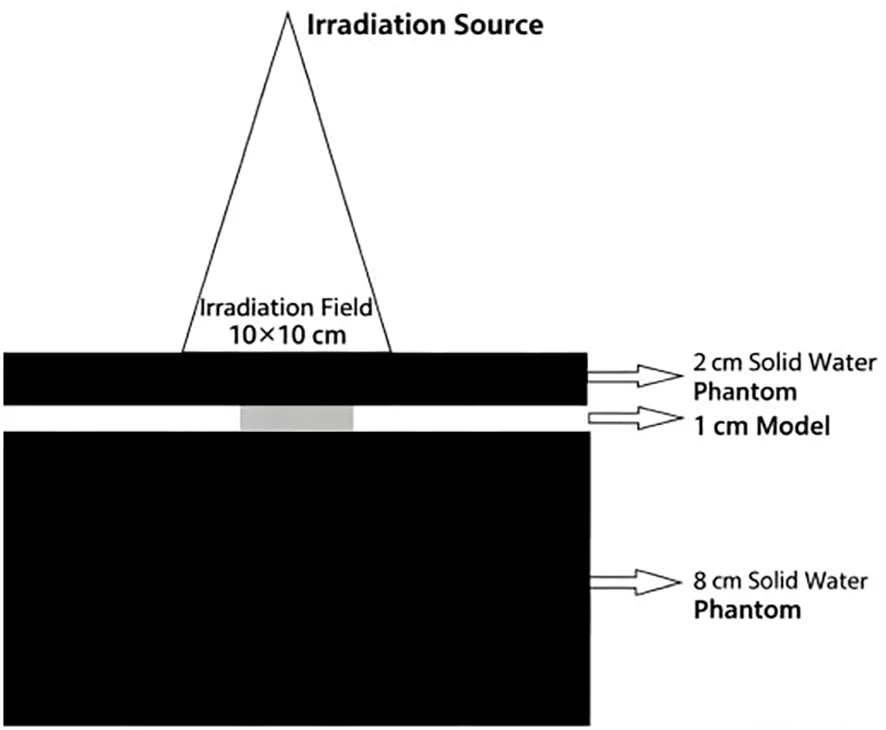
Model diagram.

A block (50×20×10mm) simulating mandibular bone was milled from polytetrafluoroethylene (PTFE) (6). The three extracted teeth were embedded into this simulated mandibular block and numbered sequentially from left to right as 1, 2, and 3, completing a partial mandibular model. This model was positioned between solid water phantoms (30×30×1 cm, IBA, Belgium): two slabs were placed superiorly to simulate buccal/labial soft tissue, and eight slabs were placed inferiorly to simulate soft tissue including the tongue, floor of the mouth, and oropharynx (**Figures 1**, **2**).

### 2.3 Tooth preparation

**Figure 3:**
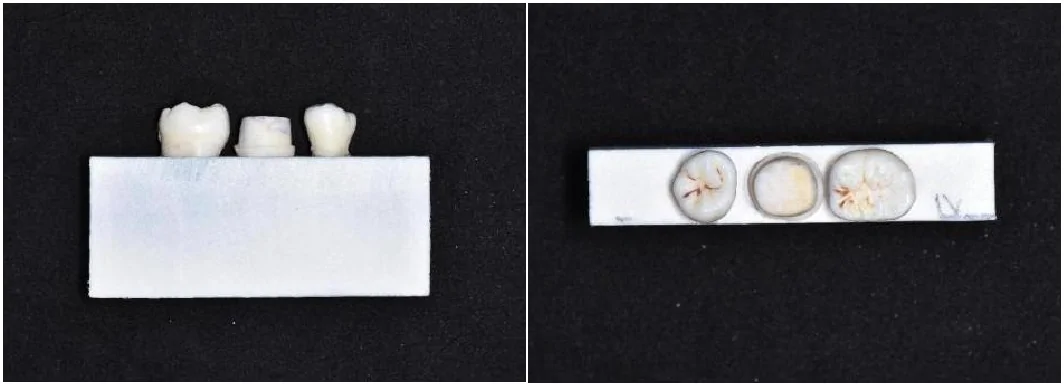
Tooth preparation.

Tooth 2 was prepared by an experienced prosthodontist (>10 years of clinical experience) using a high-speed handpiece. A 1 mm chamfer finish line was established on all axial walls. A 1 mm deep groove was prepared in the central fossa to guide uniform reduction. The tooth was prepared in a box-shaped form with rounded line angles, a convergence angle of 4–6°, and a flat occlusal surface (**Figure 3**).

### 2.4 Fabrication of full-crown restorations

Following calibration, the prepared tooth was scanned using an intraoral scanner (Trios3, 3Shape, Denmark). The Standard Tessellation Language (STL) data were exported and imported into dental CAD software (DentalCAD 3.0, Exocad, Germany). A restoration was designed based on the anatomy of a mandibular first molar. The design data were then imported into CAM software (HyperDENT, Follow Me!, Germany) for nesting and machining. Full-crown restorations were fabricated from five materials: zirconia (Meiying, Upcera, China), lithium disilicate glass-ceramic (IPS e.max, Ivoclar, Liechtenstein), resin-matrix ceramic (Lava™ Ultimate, 3M, USA), polyetheretherketone (PEEK; Biopeak, Dentsply Sirona, China), and polymethyl methacrylate (PMMA; Meijingci, Hugel, China).

**Figure 4:**
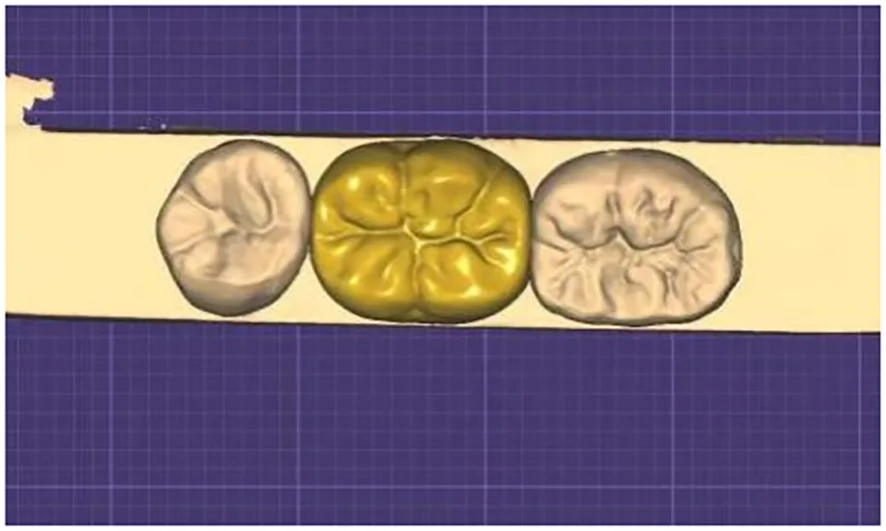
Morphological design of restoration.

**Figure 5:**
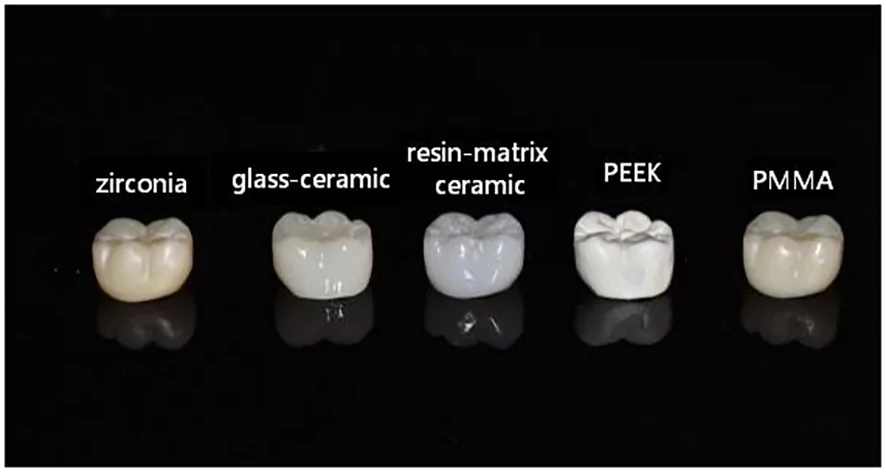
Processing of completed restorations.

The occlusal thickness and axial wall thickness of all crowns were standardized at 1.0 mm as designed in the CAD software (DentalCAD 3.0, Exocad), and a uniform cement space of 50 μm was set for all restorations. The crowns were passively seated on the prepared tooth without cementation, allowing for repeated removal and replacement of different crowns on the same preparation to ensure consistent positioning across scans; passive fit was verified visually and by tactile examination. The intraoral scanner (Trios 3, 3Shape) was calibrated according to the manufacturer’s protocol before each scanning session, with calibration verification performed using the manufacturer-supplied calibration block. All restorations were fabricated using a 5-axis milling machine (HyperDENT, Follow Me)! with a reported positioning accuracy of ±10 μm and a minimum tool diameter of 0.6 mm (**Figures 4**, **5**).

### 2.5 Model Scanning

The correctly positioned model was scanned using a dual-layer detector spectral CT (IQon Spectral CT, Philips, Netherlands), covering its entire height. Scanning parameters: Tube voltage 120 kVp; tube current fixed at 200 mA; collimator width 64 × 0.625 mm; rotation time 0.33 s/rotation; pitch 0.3; display field of view (FOV) 30 × 30 cm; matrix 512 × 512.

Reconstruction parameters: Images were reconstructed using Philips’ iterative reconstruction technique (IDose^4^) with metal artifact reduction (O-MAR) at a high energy level of 200 keV. The IDose level was set to 4. All images were reconstructed with a slice thickness and interval of 0.8 mm using a bone window (window level 500 HU, window width 2000 HU).

**Figure 6:**
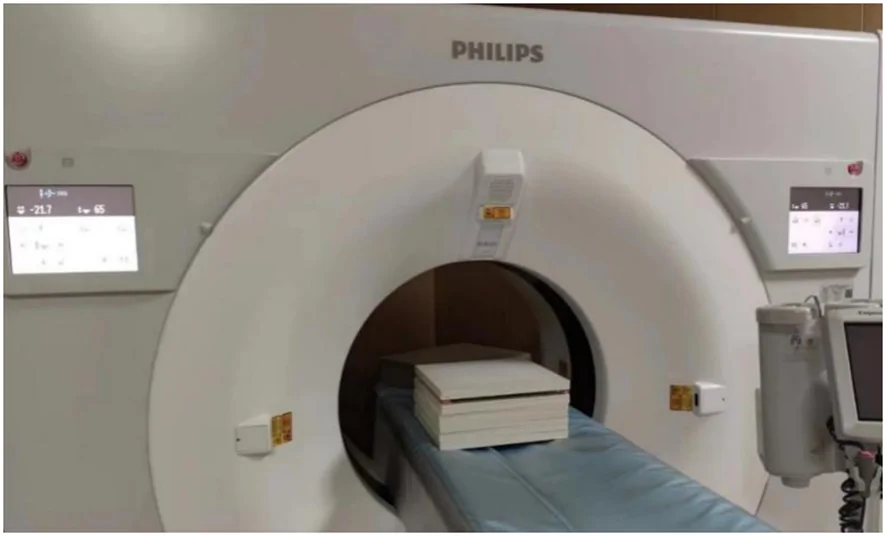
Model layout diagram.

**Figure 7:**
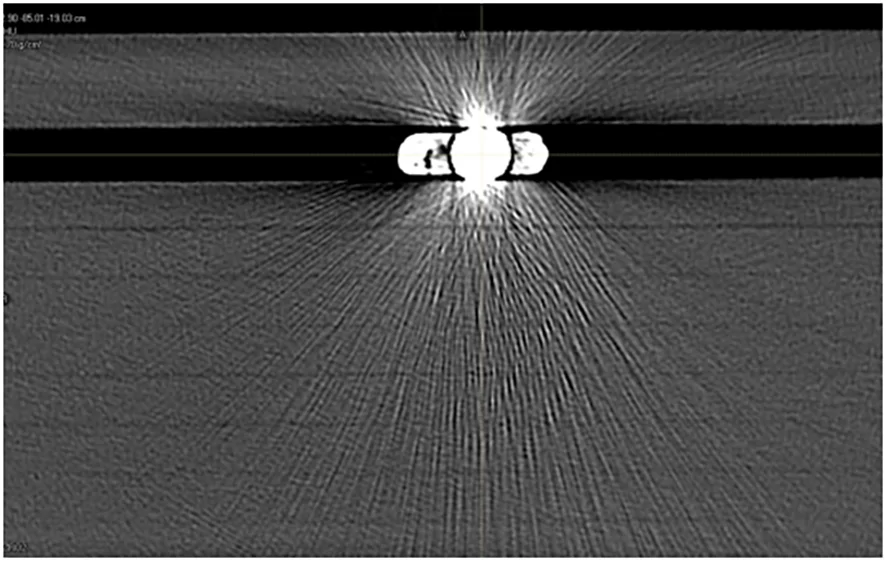
Artifact CT image (ZrO_2_).

**Figure 8:**
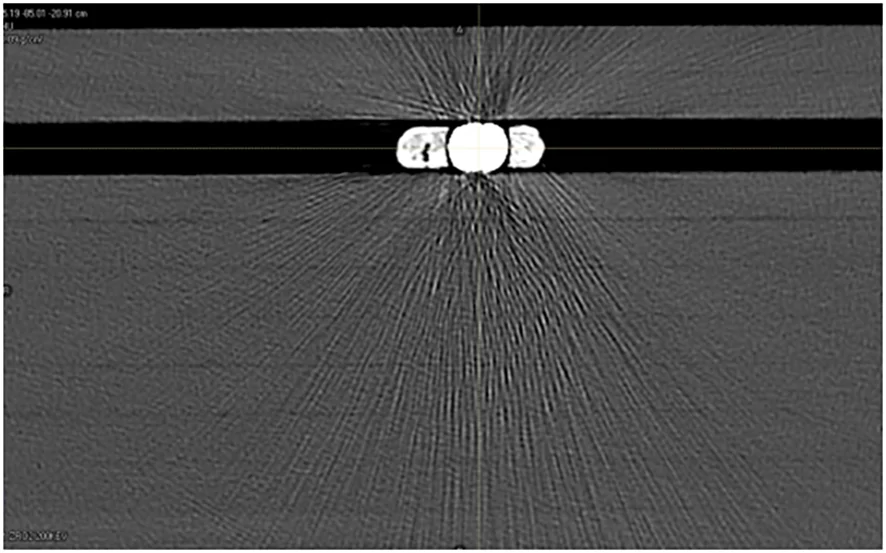
De-artifact CT image (ZrO_2_).

First, the natural tooth model (control) was scanned. Subsequently, each of the five different full-crown restorations was sequentially placed on tooth 2 and scanned. After all scans, both standard (non-artifact-reduced) and artifact-reduced CT images were exported in DICOM format, resulting in 12 scan datasets (6 standard and 6 artifact- reduced) (**Figures 6**–**8**).

### 2.6 Establishment of CT number-to-electron density curve

**Figure 9:**
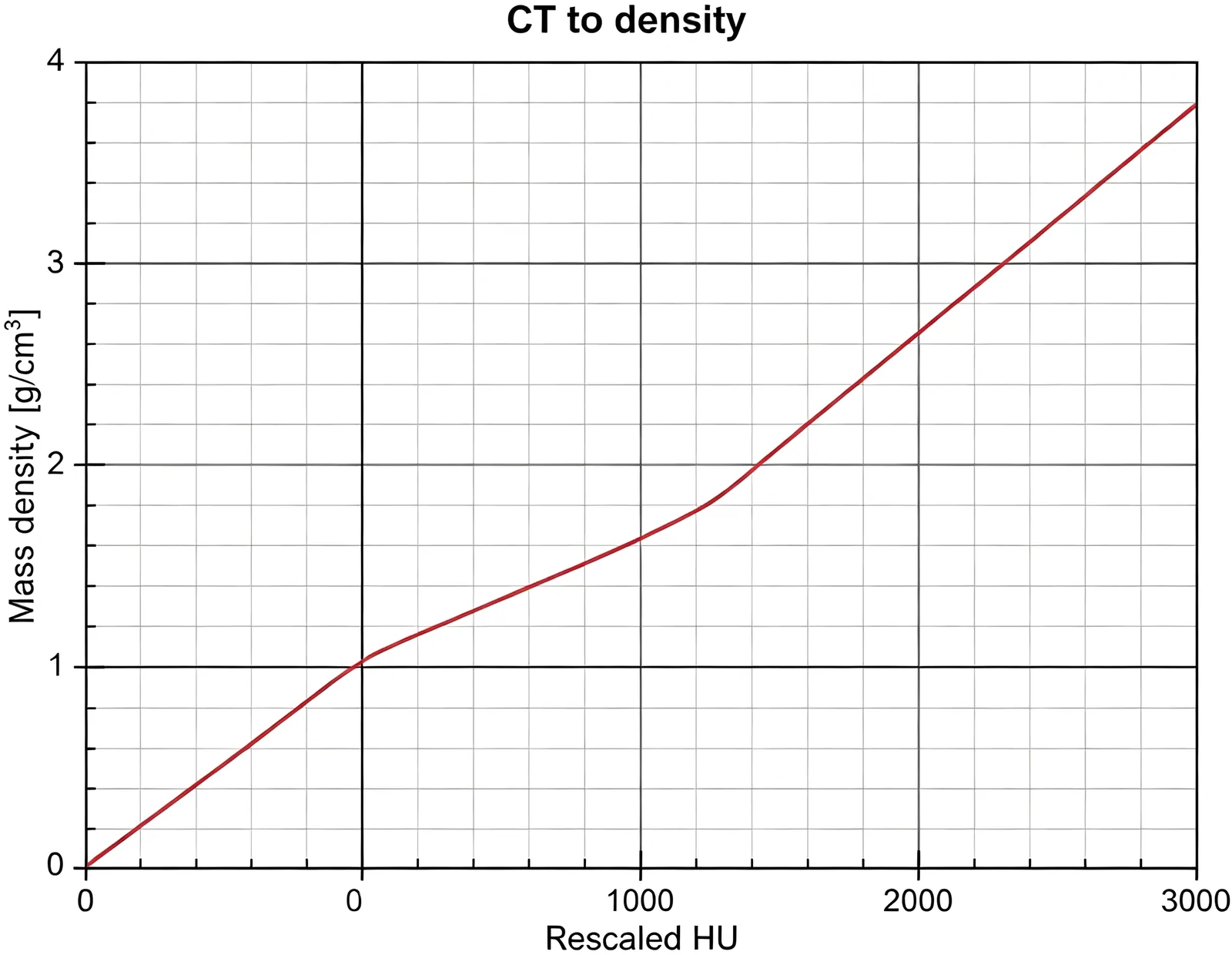
CT to ED Curve.

A CT scan was performed on an electron density phantom containing inserts of known electron densities simulating human tissues. The acquired images were imported into a CCC algorithm-based radiotherapy planning system (TPS; RayStation 9.0A, RaySearch, Sweden). Within the TPS, the CT numbers corresponding to the different tissue-equivalent inserts were correlated with their known electron densities to establish a CT number-to-electron density conversion curve (**Figure 9**).

### 2.7 Dose distribution calculation using CCC algorithm

**Figure 10:**
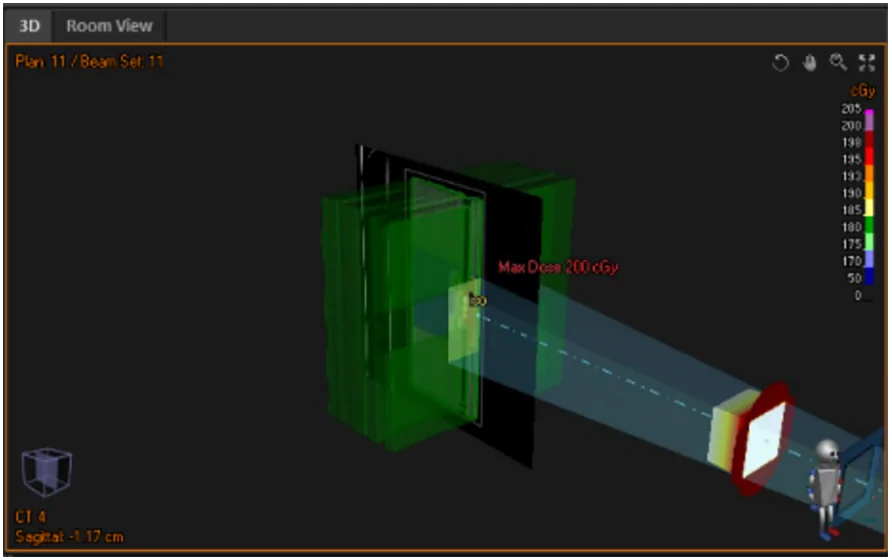
Simulation of irradiation under linear accelerator in Raystation 9.0A system.

**Figure 11:**
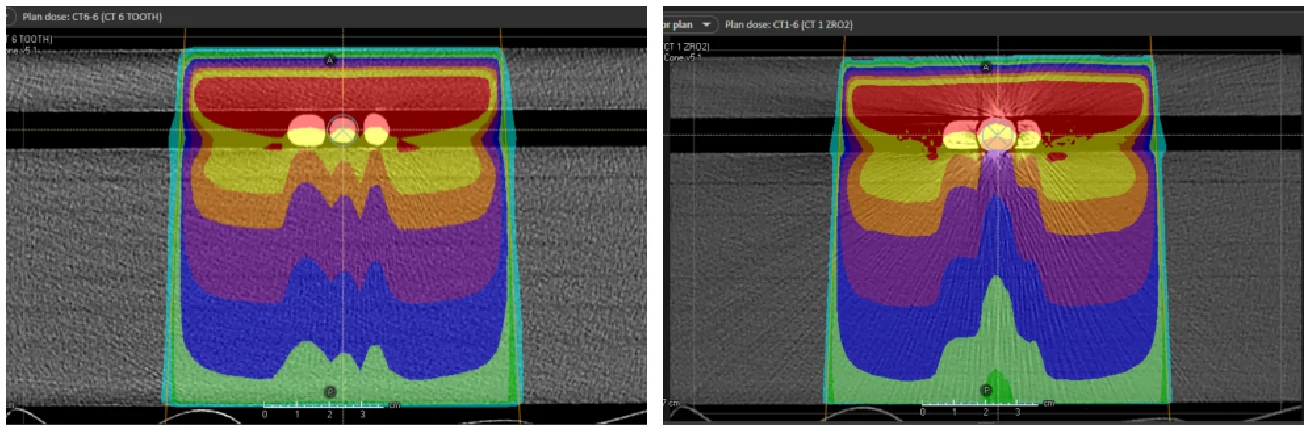
CCC algorithm 6 MV photon beam dose distribution of artifact group (Examples).

A senior radiotherapy physicist (>10 years of experience) imported all 12 scan datasets into the CCC algorithm-based TPS. A Varian medical linear accelerator (Trilogy, Varian, USA) treatment head was simulated. Single fields of 6 MV and 10 MV photon beams were configured separately with a gantry angle of 0°, 200 Monitor Units (MU), a field size of 10 × 10 cm, and a source-to-surface distance (SSD) of 100 cm. Dose distribution was calculated. With the central axis aligned to the center of the clinical crown of tooth 2, dose values were recorded at depths of 0.00, 0.25, 0.50, 0.75, 1.00, 1.25, 1.50, 1.75, 2.00, 2.25, 2.50, 2.75, and 3.00 cm from the exit surface of the restoration (defined as 0 cm). This yielded 24 radiotherapy plans (**Figures 10**, **11**).

### 2.8 Statistical analysis

The experiment was conducted in triplicate. For each replicate, 12 spectral CT image datasets were acquired, followed by radiotherapy treatment planning and dose measurement; for each set of images, point doses at 13 sites below the prosthesis were measured. The dose values obtained from the three replicates were averaged.

IBM SPSS Statistics 26.0 was used for analysis. The Shapiro-Wilk test assessed data normality. Levene’s test assessed homogeneity of variance (using the mean for normal data and the median for non-normal data). A paired samples t-test (α=0.05) analyzed dose values before and after artifact reduction for each material group. Dunnett’s t-test (α=0.05) compared dose values between each experimental material group and the natural tooth control group under identical conditions. The Tukey HSD test (α=0.05) analyzed the absolute dose difference (|non-artifact-reduced dose -artifact-reduced dose|) for each material group. Results were interpreted to provide guidance for material selection in fixed dental prostheses for radiotherapy patients.

## 3 Results

The dual-layer detector spectral CT scans produced six standard and six O-MAR+200 keV artifact-reduced CT image sets. Using the CCC algorithm in the TPS with 6 MV and 10 MV beams, dose distributions were calculated for both image types, generating 24 datasets for statistical analysis.

### 3.1 For 6 MV photon beam (CCC algorithm):

Paired samples t-tests comparing pre-and post-artifact reduction for all six groups (natural tooth, zirconia, glass-ceramic, resin-matrix ceramic, PEEK, PMMA) showed significant differences (all P < 0.001), indicating that artifacts affect radiotherapy dose distribution.

One-way ANOVA on the standard (non-artifact-reduced) image data showed significant differences among material groups (F = 6.32, P<0.001). Dunnett’s t-test revealed a significant difference only between the zirconia group and the natural tooth group (P<0.001). No significant differences were found between the natural tooth group and the glass-ceramic, resin-matrix ceramic, PEEK, or PMMA groups.

One-way ANOVA on the artifact-reduced image data showed no significant differences among groups (F = 0.58, P = 0.716).

One-way ANOVA on the absolute dose differences (pre-vs. post-artifact reduction) showed significant differences among material groups (F = 604.7, P<0.001). Tukey HSD test results indicated the following hierarchy of artifact influence on dose distribution: Zirconia (14.38) > Resin-matrix ceramic (6.92) > Glass-ceramic (5.31) > PEEK (2.92) > PMMA(2.23). PEEK and PMMAgroups showed no significant difference from the natural tooth group, but differed significantly from the zirconia, resin-matrix ceramic, and glass-ceramic groups (**Table 1**).

**Table 1:** Dose measurements for each material under a 6 MV photon beam(cGy).

Group	$\bar{x}$ ± s (A) without artifact reduction (B) with artifact reduction	Percentage dose error(%)	Maximum dose deviation
Tooth	(A) 179.69 ± 8.76	1.44%	3
	(B) 182.31 ± 8.35		
Zirconia	(A) 162.69 ± 9.56	8.13	15
	(B) 177.08 ± 8.74		
Glass-ceramic	(A) 173.77 ± 8.98	2.97	6
	(B) 179.08 ± 8.85		
Resin-matrix ceramic	(A) 173.31 ± 9.24	3.84	8
	(B) 180.23 ± 8.81		
PEEK	(A) 177.62 ± 8.81	1.62	3
	(B) 180.54 ± 8.68		
PMMA	(A) 179.08 ± 8.91	1.23	3
	(B) 181.31 ± 8.57		

### 3.2 For 10 MV photon beam (CCC algorithm):

Paired samples t-tests again showed significant differences pre- and post-artifact reduction for all groups (all P < 0.001).

One-way ANOVA on standard image data showed significant differences among groups (F = 5.14, P<0.001). Dunnett’s test confirmed a significant difference only between the zirconia and natural tooth groups (P<0.001).

One-way ANOVA on artifact-reduced image data showed no significant differences (F = 0.41, P = 0.842).

One-way ANOVA on absolute dose differences showed significant differences (F = 614.3, P<0.001). Tukey HSD test results indicated the following hierarchy of artifact influence on dose distribution: Zirconia (11.54) > Resin-matrix ceramic (5.54) > Glass-ceramic (4.54) > PEEK (2.00) > PMMA (1.85). PEEK, PMMA, and natural tooth groups showed no significant differences among themselves but differed significantly from the zirconia, resin-matrix ceramic, and glass-ceramic groups (**Table 2**).

**Table 2:** Dose measurements for each material under a 10 MV photon beam(cGy).

Group	$\bar{x}$ ± s (A) without artifact reduction (B) with artifact reduction	Percentage dose error(%)	Maximum dose deviation
Tooth	(A) 191.00 ± 7.79	1.04	3
	(B) 193.00 ± 7.51		
Zirconia	(A) 177.69 ± 8.11	6.10	12
	(B) 189.23 ± 8.03		
Glass-ceramic	(A) 186.46 ± 8.35	2.38	5
	(B) 191.00 ± 7.79		
Resin-matrix ceramic	(A) 186.38 ± 8.22	2.89	6
	(B) 191.92 ± 7.82		
PEEK	(A) 189.92 ± 7.82	1.04	3
	(B) 191.92 ± 7.66		
PMMA	(A) 190.85 ± 7.69	0.96	2
	(B) 192.69 ± 7.50		

## 4 Discussion

Research on the impact of dental prostheses on radiotherapy dose distribution has historically focused on metallic restorations. Previous studies using Monte Carlo simulations (7) and clinical dose calculations (8)have demonstrated that metallic materials can cause significant dose perturbations and increase the risk of complications such as oral mucositis. Consistent with the trend observed in prior research linking dose effects to electron density (7), the current study found that among non-metallic materials, zirconia—with its relatively high electron density—produced the most substantial artifact-related dose discrepancies. Our use of the CCC algorithm did not detect significant backscatter dose enhancement, which may be attributable to the generally lower electron densities of the selected non-metallic materials compared to metals. This suggests a potential clinical advantage in reducing mucosal toxicity.

The accurate conversion of CT numbers (HU) to electron density (ED) is fundamental for precise TPS calculations (9). The presence of high-atomic-number materials can distort this conversion due to the predominance of the photoelectric effect (10), leading to an overestimation of CT numbers and consequently assigned ED (11). In this study, the ED for zirconia was capped at the system maximum (3.79 g/cm³), implying that its true dose-disturbing effect is likely greater than reported here. This highlights a critical limitation of standard TPS conversion curves when dealing with very high-density materials.

Artifacts from dental prostheses represent another major source of dose calculation error. Conventional CT struggles with beam-hardening artifacts, particularly around high-density objects. The dual-layer spectral CT used here, combining virtual monochromatic imaging (VMI) and dedicated metal artifact reduction (O-MAR) algorithms (12, 13), allowed for a direct comparison of dose calculations with and without artifact correction. Our findings underscore that the total impact of a prosthesis on radiotherapy arises from both its physical attenuation properties (related to density and atomic number) and its propensity to generate imaging artifacts, which can lead to inaccurate target delineation and ED assignment.

The statistical analysis clearly delineated the performance of the tested materials. While zirconia significantly altered dose distribution even after artifact reduction, the other four materials (lithium disilicate glass-ceramic, resin-matrix ceramic, PEEK, and PMMA) showed no statistically significant difference from natural teeth in artifact-corrected plans. However, analysis of the absolute dose differences caused by artifacts themselves revealed a gradient: zirconia, resin-matrix ceramic, and glass-ceramic caused significantly greater artifact-induced dose inaccuracies than natural teeth, whereas PEEK and PMMA did not. The stronger penetrating power of 10 MV beams, resulting in generally lower absolute dose differences compared to 6 MV, is consistent with known beam physics.

Zirconia, despite its excellent mechanical properties and aesthetics (14–16), possesses imaging characteristics akin to metals due to the high atomic number of zirconium. Its significant artifact generation and high electron density severely compromise radiotherapy planning accuracy. Therefore, it is not recommended for fixed prostheses in head and neck cancer patients undergoing radiotherapy-a point often overlooked in clinical practice.

Lithium disilicate glass-ceramic combines favorable aesthetics with good mechanics (17, 18). While its dose distribution post-artifact-correction was equivalent to natural teeth, its artifact impact was measurably higher. Given its overall clinical performance, it remains a suitable choice for definitive restorations in this patient population.

Resin-matrix ceramics (e.g., Lava™ Ultimate) offer a balance of machinability, aesthetics, and mechanical properties (19–21). Their behavior in this study was similar to lithium disilicate glass-ceramic, making them another viable option for definitive single crowns, particularly in the anterior and premolar regions.

PEEK exhibits outstanding biocompatibility, a low elastic modulus similar to bone, and minimal imaging interference (22, 23). Although its adhesion and aesthetics require special consideration (24, 25), its negligible impact on both dose distribution and artifact generation makes it an excellent material for long-term provisional or definitive prostheses, especially for patients with metal allergies.

PMMA, the traditional provisional material, shares the low artifact profile of PEEK due to its low density (26). While its mechanical and antimicrobial properties can be inferior (27), modern composites with nanofillers show promise for improvement (28, 29). Its minimal impact on radiotherapy dose supports its continued use for provisional restorations.

In radiation oncology, the generally accepted tolerance for dose calculation accuracy is within ±3-5% of the prescribed dose, as recommended by ICRU Report 83. Applying this benchmark to our findings: Zirconia produced dose errors of 8.13% (6 MV) and 6.10% (10 MV), exceeding the 5% tolerance, indicating clinical unacceptability. In contrast, resin-matrix ceramic (3.84% at 6 MV, 2.89% at 10 MV) and glass-ceramic (2.97% at 6 MV, 2.38% at 10 MV) were within or close to the 5% limit, supporting their cautious use. PEEK and PMMA showed errors well below 3%, making them the safest options.

The present findings are consistent with the growing emphasis on clinically implemented RayStation workflows for assessing plan quality and deliverability. Recent studies using RayStation have shown that dosimetric evaluation should not be limited to dose-volume indices alone, but should also consider delivery accuracy, beam characteristics, and the practical performance of the treatment-planning workflow. In this context, our study extends previous RayStation-based investigations by focusing on a different but clinically important source of uncertainty: dental-prosthesis-related CT artifacts and their influence on HU-to-electron-density conversion and CCC dose calculation. By comparing artifact- containing and artifact-reduced CT datasets under 6 MV and 10 MV photon beams, the present work provides practical evidence for selecting dental prosthetic materials that are more compatible with head-and-neck radiotherapy planning.

In summary, this study challenges the common assumption that all-ceramic restorations are benign in the radiotherapy context. Zirconia, in particular, can significantly disrupt treatment planning. Among the non-metallic alternatives, lithium disilicate glass-ceramic and resin-matrix ceramic are recommended for definitive restorations, while PEEK and PMMA are ideally suited for provisional crowns due to their minimal artifact generation. This study is limited by its focus on single crowns and the TPS-imposed upper limit on the assigned electron density for zirconia, which may underestimate its true effect. Future research should investigate multi-unit fixed dental prostheses (bridges) and implant-supported prostheses. Our model used PTFE to simulate mandibular bone and solid water phantoms for soft tissue, which, while standardized, cannot fully replicate the heterogeneous tissue composition and complex anatomy of actual patients. This may affect the magnitude but not the relative ranking of material-induced dose errors. The simplified geometry does not account for irregular tissue contours, air cavities (e.g., paranasal sinuses, oral cavity), or neighboring critical structures. Future studies using patient-specific phantoms or retrospective clinical datasets are warranted. No Monte Carlo validation: While the CCC algorithm is widely used and validated for heterogeneous media, Monte Carlo simulation remains the gold standard for dose calculation in the presence of high-density materials. We acknowledge that MC validation would strengthen our findings and plan to pursue this in future work. Only one TPS algorithm evaluated: Our results are specific to the RayStation 9.0A implementation of the CCC algorithm. Different TPS platforms or algorithm versions (e.g., Acuros XB, Monte Carlo) may yield different results. We encourage multi-platform validation. No experimental dosimetry validation: We did not perform physical dose measurements using ionization chambers, films, or other detectors. While our CT-to-ED calibration followed standard clinical protocols, experimental validation would provide an independent check of our computational results. No comparison with CBCT-based planning: CBCT is increasingly used for adaptive radiotherapy. However, CBCT suffers from more severe scatter and artifact issues than spectral CT, and its Hounsfield unit calibration is less stable. Our study focused on spectral CT; CBCT-based planning represents a distinct and important direction for future investigation.

## 5 Conclusions

Zirconia exhibits high electron density and generates significant CT artifacts, severely compromising the accuracy of radiotherapy dose calculations. It is not recommended for fixed dental prostheses in patients undergoing head and neck radiotherapy. Lithium disilicate glass-ceramic, resin-matrix ceramic, PEEK, and PMMA demonstrate dose distributions statistically equivalent to natural tooth controls in artifact-corrected plans and are considered suitable for clinical use. However, regarding the magnitude of artifact-induced dose inaccuracies, lithium disilicate glass-ceramic and resin-matrix ceramic produce a greater effect than natural teeth, whereas PEEK and PMMA do not. Therefore, based on a combination of material properties and radiotherapy compatibility, PEEK and PMMA are highly recommended for provisional crowns, while lithium disilicate glass-ceramic and resin-matrix ceramic are recommended for definitive restorations in this patient population.

## Data Availability

The original contributions presented in the study are included in the article/supplementary material. Further inquiries can be directed to the corresponding author.
